# GLP‐1 Receptor Agonists Post‐Bariatric Surgery (GRABS) Trial: Rationale, Design and Baseline Characteristics

**DOI:** 10.1111/dom.71320

**Published:** 2026-09-08

**Authors:** J. M. Samuels, C. R. Williams, M. B. Patel, W. H. Self, L. M. Funk, C. Roumie, M. D. Spann, C. J. Northup, F. Ye, R. Irlmeier, I. Sohn, Y. Zhu, K. Niswender

**Affiliations:** ^1^ Department of Surgery Vanderbilt University Medical Center Nashville Tennessee USA; ^2^ Vanderbilt Medical Student Research Training Program Vanderbilt University Medical Center Nashville Tennessee USA; ^3^ Department of Emergency Medicine Vanderbilt University Medical Center Nashville Tennessee USA; ^4^ Department of Surgery University of Wisconsin‐Madison Madison Wisconsin USA; ^5^ Department of Surgery William S. Middleton VA Madison Wisconsin USA; ^6^ Department of Surgery William S. Middleton Veterans Health Administration Hospital Madison Wisconsin USA; ^7^ Tennessee Valley VA Health Care System Nashville Tennessee USA; ^8^ Department of Public Health Sciences, Sylvester Comprehensive Cancer Center University of Miami Miami Florida USA; ^9^ Department of Biostatistics Vanderbilt University Medical Center Nashville Tennessee USA; ^10^ Department of Medicine, Division of Diabetes, Endocrinology and Metabolism VUMC Nashville Tennessee USA

**Keywords:** bariatric surgery, GLP‐1 receptor agonist, GRABS trial, multimodal obesity treatment, obesity management medication, weight recurrence

## Abstract

**Aims:**

No studies have investigated the synergistic effectiveness of Glucagon‐Like Peptide 1 Receptor Agonists (GLP1RAs) in patients with persistent obesity following bariatric surgery. The GLP‐1 Receptor Agonists Post‐Bariatric Surgery (GRABS) Trial aims to investigate the effectiveness of tirzepatide (TRZ) versus standard of care (SOC) after Roux‐en‐Y gastric bypass (RYGB) in patients with persistent obesity (body mass index [BMI] ≥ 30 kg/m^2^).

**Materials and Methods:**

This randomized, open‐label proof‐of‐concept trial compares 24 weeks of TRZ with SOC versus SOC alone after RYGB. The primary outcome is total percent weight change from baseline to 24 weeks after trial initiation. Patients aged 25–65 with BMI ≥ 30 kg/m^2^ enroled 12 months after RYGB are randomized 1:1 to 24 weeks of TRZ or SOC. Exclusion criteria include: type 1 diabetes, type 2 diabetes treatment other than metformin, conversion to RYGB from prior bariatric surgery, and GLP1RA use in the past 3 months.

**Results:**

The study enroled 33 patients, with 5 withdrawals prior to completing a baseline visit, resulting in a final sample of 28 patients. The final cohort is 82% female, 75% white, median age 42.0 years, pre‐surgery BMI of 48.3 kg/m^2^, baseline BMI at randomization of 36.0 kg/m^2^ and baseline weight of 95.6 kg.

**Conclusions:**

GRABS is the first randomized trial to investigate GLP1RA effectiveness in patients with persistent obesity following RYGB, with the potential to inform synergistic adjuvant strategies to enhance surgical weight loss.

**Trial Registration:**

ClinicalTrials.gov identifier: NCT06162715

## 1 Introduction

Obesity is a chronic disease affecting more than 40% of adults in the United States and is a leading driver of cardiovascular disease, type 2 diabetes, metabolic dysfunction–associated steatotic liver disease, obstructive sleep apnea and several malignancies [1, 2, 3]. Sustained weight reduction lessens the morbidity and mortality attributable to obesity, with the magnitude of clinical benefit broadly tracking the magnitude and durability of weight loss achieved, regardless of the modality [4, 5, 6, 7, 8]. This evidence has motivated the development of progressively more effective single‐modality treatments and, increasingly, the question of how much additional benefit might be realized when those treatments are used in combination.

Bariatric surgery remains the most effective single treatment for severe obesity. Roux‐en‐Y gastric bypass (RYGB) produces approximately 25%–30% total body weight loss at long‐term follow‐up, with durable improvements in cardiometabolic comorbidities and reductions in all‐cause mortality [9, 10, 11]. However, weight loss response is heterogeneous. A significant minority of patients experience suboptimal initial weight loss or clinically significant weight regain [12], and even among patients with an expected surgical response, many do not reach a body mass index (BMI) below the threshold for obesity [13, 14]. Persistent obesity after an otherwise successful operation leaves measurable residual cardiometabolic risk and represents an unmet need that surgery alone cannot address [15].

The recent emergence of highly effective anti‐obesity medications has changed what is pharmacologically achievable. In adults with obesity who have not undergone bariatric surgery, glucagon‐like peptide‐1 receptor agonists (GLP1RAs) and the dual glucose‐dependent insulinotropic polypeptide (GIP) receptor agonist/GLP1RA tirzepatide (TRZ) produce 15%–22% mean body weight reduction in pivotal trials, with concurrent improvement in blood pressure, lipids, glycemia, hepatic steatosis [16, 17, 18], and cardiovascular event reduction in patients with established disease [19]. In post‐bariatric populations, two randomized trials of liraglutide have informed practice: GRAVITAS evaluated liraglutide 1.8 mg for persistent or recurrent type 2 diabetes after metabolic surgery [20], and BARI‐OPTIMISE evaluated liraglutide 3.0 mg in patients with ≤ 20% total body weight loss (TWL) following surgery [21]. Both trials enroled patients on the basis of an inadequate surgical response and positioned pharmacotherapy as rescue. Most recently, the BARI‐STEP trial demonstrated the benefit of GLP1RA as rescue therapy with semaglutide in patients with ≤ 20% TWL following bariatric surgery [22]. Observational data with semaglutide and TRZ in similar post‐bariatric non‐responder cohorts have shown comparable directional effects [23, 24, 25].

Whether GLP1RAs and related medications can be used proactively, after an expected surgical response, to augment weight loss and drive a meaningful fraction of patients towards remission of obesity has not been tested in a randomized trial. This question is the foundation of a treat‐to‐target paradigm for obesity care, in which a defined end‐organ goal directs the stepped, combined use of available therapies. Such a framework is standard in the management of hypertension, dyslipidemia and type 2 diabetes but has not been operationalized in obesity. The combination of bariatric surgery and incretin therapy raises this possibility for the first time.

Despite a decade of evidence establishing the efficacy of GLP1RA for weight loss, the anatomic origin of their characteristic GI adverse effects, including nausea, vomiting, early satiety and bloating, remains unresolved: whether these adverse symptoms arise peripherally, through vagally‐mediated delay of gastric emptying, or centrally, through agonism at the brainstem area postrema and nucleus tractus solitarius [26, 27, 28]. RYGB provides a natural dissociation of these mechanisms. Because the gastric pouch empties rapidly, intestinal transit is accelerated, and the remnant stomach and pylorus are excluded from the path of ingested nutrients [29, 30], the substrate for emptying‐dependent symptoms is largely bypassed while central pro‐emetic receptor populations remain intact and accessible to the circulating drug. Persistence of symptoms in the altered RYGB anatomy would implicate a central origin, whereas attenuation relative to non‐surgical populations would support a peripheral, emptying‐dependent contribution. This positions the GRABS trial to assess how TRZ impacts GI symptomatology after RYGB.

Herein is a presentation of the clinical trial design, objectives, endpoints and baseline characteristics of the GLP‐1 Receptor Agonists Post‐Bariatric Surgery (GRABS) proof‐of‐concept trial, the first randomized clinical trial of GLP1RA‐class agents at any time after RYGB, specifically in patients with persistent obesity despite expected surgical weight loss.

## 2 Materials and Methods

### 2.1 Study Design

This is a single‐centre, open‐label, parallel randomized trial. Participants are enroled 12 months after undergoing RYGB and are randomized to receive 24 weeks of TRZ in addition to the standard of care (SOC), or continue with the SOC dietary and supportive interventions alone implemented after bariatric surgery. After 24 weeks, participants in the intervention arm discontinue TRZ, and both groups remain on study (Figure 1a). Due to initial challenges with patient dropout in the control arm (*N* = 4 vs. 1 in the intervention arm), the study includes an amended protocol so that after the initial 24 weeks, participants in the control arm cross over to the intervention arm to receive 24 weeks of TRZ (Figure 1b).

**FIGURE 1:**
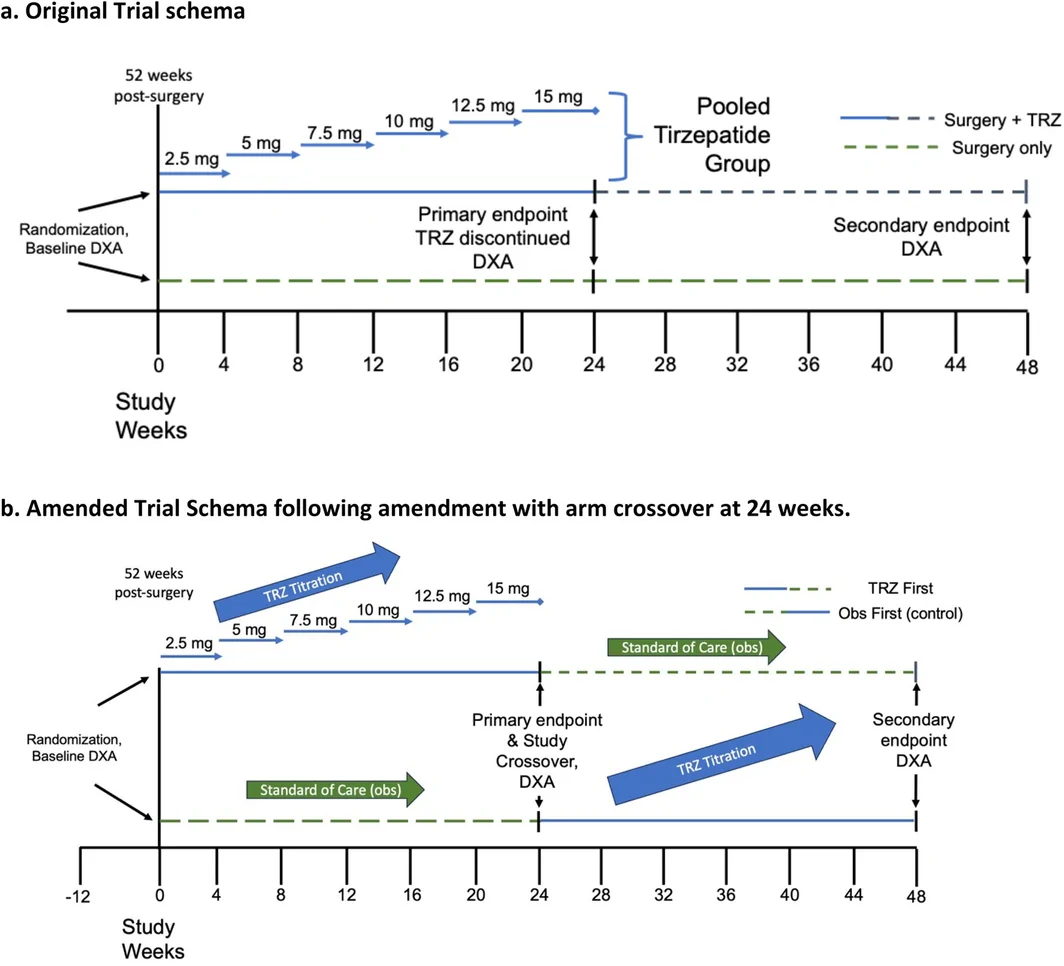
(a) Original trial schema. (b) Amended trial schema following amendment with arm crossover at 24 weeks. DXA, dual‐energy X‐ray absorptiometry; Obs, observation; TRZ, tirzepatide.

### 2.2 Study Participants

GRABS is being conducted at a single academic medical centre, with participants recruited from the Vanderbilt University Medical Center Surgical Weight Loss Clinic. Key inclusion criteria are adults 25–65 years of age with a BMI ≥ 30 kg/m^2^ at screening and a history of primary RYGB performed 12 ± 2 months prior to enrolment. Key exclusion criteria include any history of revisional bariatric surgery or a primary bariatric procedure other than RYGB; type 1 diabetes or type 2 diabetes requiring antidiabetic therapy beyond stable metformin; and use of any GLP1RA ≤ 90 days of screening. Additional exclusions reflect the established safety profile of TRZ. The complete list of eligibility criteria is provided in Table 1. The trial is being conducted in accordance with Good Clinical Practice guidelines, and the protocol was approved by the Vanderbilt University Medical Center Institutional Review Board (IRB# 240083). All participants provide written informed consent electronically prior to any study procedures. The trial is registered at ClinicalTrials.gov under the identifier NCT06162715.

**TABLE 1:** Key eligibility criteria.

Inclusion criteria
Adults aged 25–65 years
Body mass index ≥ 30 kg/m^2^ at screening
History of primary Roux‐en‐Y gastric bypass performed approximately 12 months prior to enrolment
Exclusion criteria
Consent and adherence
Inability or unwillingness to provide informed consent or to comply with the study protocol
Diabetes and glucose‐lowering therapy
Type 1 diabetes
Use of antidiabetic agent other than metformin, within 90 days prior to screening (stable‐dose metformin permitted if maintained for ≥ 90 days prior to screening and held stable throughout the trial)
Surgical history
History of revisional bariatric surgery or of a primary bariatric procedure other than RYGB.
Cardiovascular
Myocardial infarction, unstable angina, stroke, or heart failure (NYHA class II or greater) within the prior year
Uncontrolled hypertension (systolic blood pressure > 150 mmHg or diastolic blood pressure > 90 mmHg)
Other medical
Personal history of pancreatitis
Personal or family history of medullary thyroid carcinoma or multiple endocrine neoplasia syndrome type 2
Untreated hyperthyroidism
History of physician‐diagnosed malignancy other than excised non‐melanoma skin cancer within the prior 5 years
History of solid organ transplant
Estimated glomerular filtration rate < 60 mL/min/1.73 m^2^ at randomization
Use of systemic glucocorticoids within 28 days prior to screening
Reproductive
Pregnancy, current lactation, or plans to become pregnant during the study period
Behavioural
Tobacco, electronic cigarette, or marijuana use within 12 months prior to enrolment
Current or self‐reported drug or alcohol use disorder

Abbreviations: NYHA, New York Heart Association; RYGB, Roux‐en‐Y gastric bypass.

Enrolment was anchored to a fixed postoperative interval (12 ± 2 months) rather than to documented weight stability. Weight nadir after RYGB typically occurs between 12 and 18 months [31] and cannot be identified prospectively at the individual level given routine postoperative follow‐up intervals, and post‐nadir weight rebound is common, such that a stability‐based entry criterion would both delay enrolment and fail to define a genuinely stable baseline. Residual surgery‐attributable weight loss after 12 months is small relative to the anticipated treatment effect, and because it is expected to occur in both arms, randomization renders it a component of the outcome shared by the two groups rather than a source of bias in the between‐arm comparison. The primary estimand is therefore the incremental effect of TRZ over the ongoing standard postoperative trajectory, which corresponds to the clinical decision the trial is intended to inform.

### 2.3 Intervention

Participants are randomized 1:1 to one of two open‐label parallel arms: an immediate‐treatment arm in which TRZ is initiated upon study entry (approximately 12 months after RYGB), or a delayed‐treatment arm in which participants receive standard post‐bariatric care alone for an initial 24‐week observation period before crossing over to TRZ. In both arms, TRZ is administered for 24 weeks. Participants in the immediate‐treatment arm complete an additional 24‐week off‐treatment observation period after TRZ discontinuation, yielding a total study duration of 48 weeks for all participants.

The trial, as initially designed, did not incorporate a crossover; the amended protocol includes delayed‐treatment crossover for the reasons described previously. This crossover occurs following completion of the primary outcome at 24 weeks and thus has no impact on the primary outcome. No scheduled crossover occurs prior to 24 weeks. The change additionally permits within‐trial assessment of TRZ initiated at 12 versus 18 months post‐RYGB. The trial is conducted open‐label as no commercially available placebo matches the proprietary TRZ single‐dose injector pen.

TRZ is administered as a once‐weekly subcutaneous injection beginning at 2.5 mg and escalating in 2.5 mg increments every 4 weeks, consistent with the titration schedule used in the SURMOUNT trials [17], to a target dose of 15 mg or the maximum tolerated dose. Participants who experience treatment‐intolerable gastrointestinal symptoms are first counselled on dietary modifications shown to reduce symptom burden, including consuming smaller volumes per meal, increasing the number of daily meals, and stopping intake at the onset of satiety. If symptoms persist, the protocol permits temporary interruption of the study drug followed by resumption at a reduced dose. Participants with intolerable symptoms that persist despite these measures are discontinued from the study drug. Use of additional prescription anti‐obesity medications obtained outside the study is prohibited during the treatment period. Metformin is permitted at a stable dose for glycemic control.

All participants receive the standard post‐bariatric care delivered through the Vanderbilt Surgical Weight Loss program throughout the study protocol. Dietary recommendations follow the Vanderbilt Surgical Weight Loss patient handbook and include a minimum daily protein intake of 60 g, fluid intake of at least 64 fluid ounces per day, and daily bariatric‐specific multivitamin supplementation. Participants retain access to the program's dedicated bariatric dietitians, advanced practice providers and surgeons on an as‐needed basis throughout the trial, reflecting the SOC available to patients in the first two postoperative years at our centre. This pragmatic comparator was chosen deliberately: the trial is designed to evaluate the additive effect of TRZ on SOC post‐RYGB, rather than against an intensified or study‐specific lifestyle intervention that would not reflect routine clinical practice and would limit the external validity of the findings.

### 2.4 Objectives and Outcome Measures

#### 2.4.1 Primary Objectives and Endpoint

The primary objective of GRABS is to evaluate whether TRZ initiated approximately 12 months after RYGB produces greater body weight reduction than standard post‐bariatric care alone over 24 weeks of treatment in adults with persistent obesity. We hypothesize that 24 weeks of adjuvant TRZ will produce an additional 9% TWL compared with standard post‐bariatric care. The primary endpoint is the percent change in TWL from randomization (Week 0) to Week 24, calculated as:

$$\%\text{Total Weight Loss}=\frac{\left(\text{Week}\;0\;\text{body weight}-\text{Week}\;24\;\text{body weight}\right)\;}{\text{Week}\;0\;\text{body weight}}\times 100$$

Body weight measurements are obtained at the Vanderbilt Clinical Research Center. To investigate the adverse symptomatology of TRZ in patients who have undergone RYGB, we will employ a 24‐week median Patient Assessment of Upper Gastrointestinal Symptom Severity Index (PAGI‐SYM) score as a co‐primary endpoint.

#### 2.4.2 Key Secondary Objectives and Endpoints

Key secondary objectives are organized into three domains: anthropometric changes, gastrointestinal symptomatology and adverse events. The corresponding endpoints are categorical weight loss outcomes (≥ 5%, ≥ 10% and ≥ 15% total body weight loss), body composition outcomes assessed by dual‐energy X‐ray absorptiometry, change in PAGI‐SYM sub‐scores over the 24‐week treatment period, and cardiometabolic outcomes, including glycemic, lipid and blood pressure parameters. Complete definitions, measurement timepoints and analytic methods for all secondary endpoints are provided in Table 2.

**TABLE 2:** Primary, secondary, and exploratory outcome measures.

Endpoint	Definition	Assessment timepoint
Primary outcome		
Percent total body weight loss	((Week 24 − Week 0 weight)/Week 0 weight) × 100, using in‐clinic measurements	Week 0 to Week 24
Gastrointestinal symptom burden	Adjusted mean total score on the PAGI‐SYM; 20 items across 6 subscales over the treatment period	Monthly, Week 0 through Week 24
Secondary outcomes—Timing of initiation		
12‐ vs. 18‐month initiation	Between‐group difference in % TWL after 24 weeks of tirzepatide, comparing immediate‐treatment (initiation ~12 months post‐RYGB) and delayed‐treatment (initiation ~18 months post‐RYGB, after crossover) participants.^a^	After 24 weeks of tirzepatide exposure in each arm
Secondary outcomes—Categorical weight response		
Weight‐loss responder thresholds	Proportion of participants achieving ≥ 5%, ≥ 10%, and ≥ 15% total body weight loss	Week 0 to Week 24
Secondary outcomes—Body composition (DXA)		
Body composition	Change from baseline in total fat mass, total lean mass, lean‐to‐fat mass ratio, and visceral and subcutaneous adipose tissue, measured by DXA	Week 0 to Week 24, Week 24 to Week 48
Secondary outcomes—Anthropometric		
Anthropometrics	Change from baseline in waist circumference and waist‐to‐hip ratio	Week 0 to Week 24, Week 24 to Week 48
Secondary outcomes—Cardiometabolic		
Cardiometabolic parameters	Change from baseline in HbA1c, fasting glucose, fasting insulin, fasting lipid panel (total, LDL, HDL, and VLDL cholesterol; triglycerides), and systolic and diastolic blood pressure	Week 0 to Week 24, Week 24 to Week 48
Exploratory outcomes		
PAGI‐SYM subscale scores	Change from baseline in each PAGI‐SYM subscale: heartburn/regurgitation, nausea/vomiting, postprandial fullness, bloating, upper abdominal pain, and lower abdominal pain	Monthly, Week 0 through Week 24
Post‐discontinuation symptom trajectory	Change in PAGI‐SYM score within the tirzepatide arm from end of treatment to 4 weeks after treatment discontinuation	Week 24 to Week 28

Abbreviations: %TWL, percent total weight loss; DXA, dual‐energy X‐ray absorptiometry; HbA1c, glycated haemoglobin; HDL, high‐density lipoprotein; LDL, low‐density lipoprotein; PAGI‐SYM, Patient Assessment of Upper Gastrointestinal Symptom Severity Index; RYGB, Roux‐en‐Y gastric bypass; VLDL, very low‐density lipoprotein.

^a^ Post hoc outcome comparison included after trial amendment allowing for control arm crossover.

### 2.5 Randomization Strategy

Given the small sample size and thus risk for imbalance despite randomization, the study employs Big Stick randomization method with a Maximally Tolerated Imbalance (MTI) to achieve a balanced stratification. Initially, we randomize patients 1:1 in an unrestricted manner until achieving an MTI of two [32, 33]. At that point, using a Big Stick Design to correct imbalance, randomization will be stratified by baseline BMI, sex and race/ethnicity with an attempt to balance for these factors. The randomization allocation tables are generated by the study biostatistician with no other study team members having access to these tables. However, the study is conducted open‐label given the absence of an available placebo using the proprietary injection pen.

### 2.6 Safety and Nutritional Monitoring

All participants receive standard post‐bariatric nutritional care throughout the trial, including daily bariatric‐specific micronutrient supplementation and a minimum daily protein intake of 60 g. Micronutrient laboratory surveillance follows the schedule used at our centre for all RYGB patients, at 1, 3, 6 and 12 months postoperatively and annually thereafter, consistent with ASMBS Integrated Health Nutritional Guidelines [34]. Because participants enrol 12 months after surgery, the interval of highest deficiency risk is completed under this surveillance prior to study entry. No additional micronutrient monitoring is specified by the protocol, as neither current guidelines nor prior randomized trials of GLP‐1 receptor agonist therapy after metabolic surgery define monitoring requirements for combined therapy. Treatment‐emergent gastrointestinal symptoms are captured through monthly PAGI‐SYM administration and protocol‐directed adverse event review. The protocol specifies a total weight floor: dose is reduced by 2.5 mg with reassessment at 4 weeks if BMI falls below 20 kg/m^2^, and study drug is discontinued if BMI reaches 18 kg/m^2^ or below. All serious adverse events are reported to an independent data and safety monitoring board, which reviews and adjudicates each event, including determination of relatedness to study drug, independently of the study team.

### 2.7 Statistical Analyses

The planned study sample size is 30 subjects total (15 in each arm). Utilizing conservative estimates from the SURMOUNT trial, we assume a standard deviation of 7% TWL [17]. If the true difference between arms in percent TWL at 24 weeks is 9%, we will be able to reject the null hypothesis that the population means of the intervention and control groups are equal with a probability (power) of 0.85 and an alpha of 0.05. This estimate accounts for a 25% dropout rate in each arm. A clinically meaningful difference for weight loss in this study is defined as 5%, as per the FDA guidelines [35].

We will conduct the primary analysis using a modified Intention‐to‐Treat (mITT) approach, including all randomized participants who completed baseline assessments. No outcome data will be imputed. Participants without Week‐24 outcome data will be included in the mITT analysis. We will also perform per‐protocol analyses among participants who completed baseline and 24‐week assessments.

For the primary outcome of %TWL, descriptive and graphic exploratory analysis will be conducted to show the difference in mean weight change at 24 weeks by the two treatment groups. The GI adverse symptomatology endpoint, change in PAGI‐SYM score over the 24‐week treatment period, is analysed using a linear mixed‐effects model for repeated measures of total PAGI‐SYM scores obtained monthly. The model specifies fixed effects for treatment group, visit (categorical), and the treatment‐by‐visit interactions, with adjustment for baseline PAGI‐SYM score. A participant‐level random effect is included to account for within‐participant correlation across repeated measurements. The adjusted between‐group difference at Week 24 and the time‐averaged between‐group difference across the treatment period are estimated using model‐based linear contrasts, yielding both the Week 24 endpoint effect and the overall period‐wide treatment effect with internally consistent standard errors.

Secondary outcomes, including waist circumference, waist‐to‐hip ratio, lean body mass and the ratio of lean mass to fat mass, will be analysed using similar methods to the primary outcome %TWL. Bonferroni corrections will be applied to adjust for multiple comparisons. Additional sensitivity analyses will be employed to evaluate the rigour of our findings. As a sensitivity analysis to investigate the effect of any potential residual imbalances between groups, we will use inverse probability weighting (IPW). If missing baseline covariate data exist, we will consider simple or multiple imputation for covariates, as appropriate, assuming data are missing at random; sensitivity analyses will compare results from complete‐case analyses and imputed datasets. Pre‐randomization weight trajectory (weight at surgery and at 12 months postoperatively) is reported by arm.

## 3 Results

The GRABS trial began enrolment in November 2024 and has completed enrolment as of April 2026. Data collection for the primary outcome will conclude in October 2026, and all data collection will conclude in April 2027. Thirty‐three participants were enroled, with 5 immediate withdrawals, leaving 28 for the mITT analysis set (Figure 2). In total, 18 participants were randomized to the intervention arm and 15 to the control arm. For the mITT analysis set, 17 participants were in the intervention arm and 11 were in the control arm.

**FIGURE 2:**
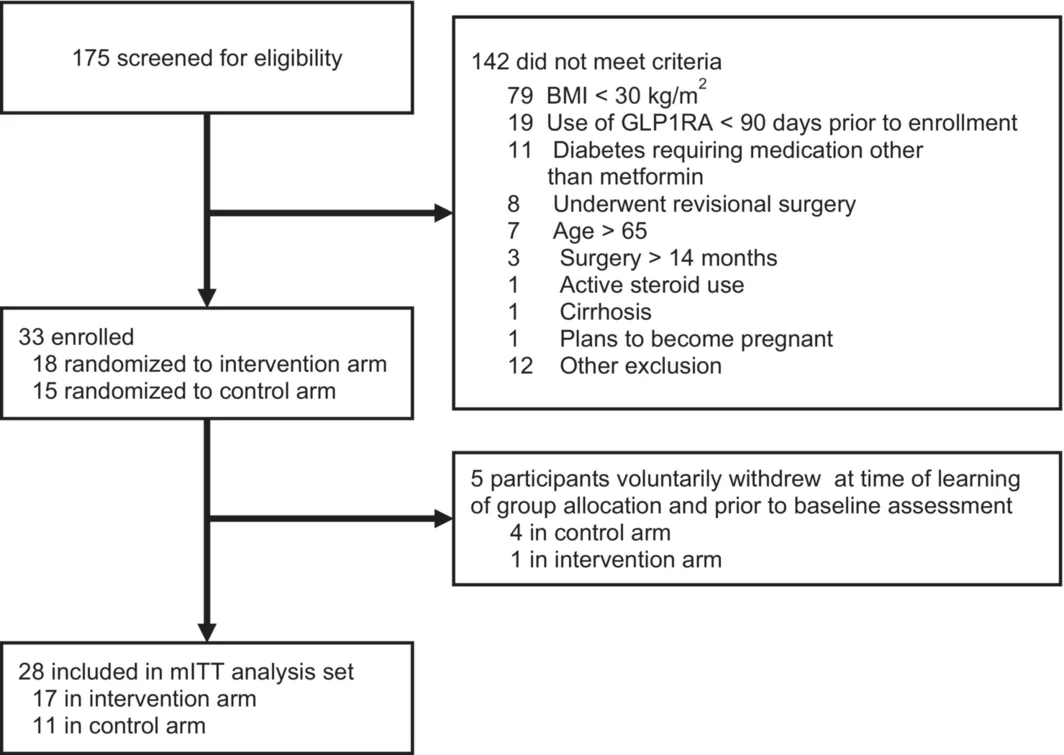
CONSORT diagram demonstrating group allocation, withdrawal, and modified Intention‐to‐Treat Sample Set.

The median age of the cohort in the mITT population was 42 years (interquartile range [IQR]: 26–50), 75% were white, 25% black, 82% female, with a median body weight of 95.6 kg (IQR: 73.6–109.1), and a median BMI of 36 kg/m^2^ (IQR: 30–40). Of pre‐existing comorbidities in the mITT cohort, 57% had hypertension, 48% obstructive sleep apnea, 22% metabolic dysfunction–associated steatotic liver disease, 22% Osteoarthritis and 4% Type 2 Diabetes. Median body weight at time of surgery (12 months prior to enrolment) was 135.3 kg (IQR: 96.0–154.0), median BMI at time of surgery was 48.3 kg/m^2^ (IQR: 39.0–53.1) and 12‐month post‐gastric bypass (pre‐enrolment) %TWL of 25% (IQR: 21.4%–30.3%). Group demographics are provided in Table S1.

## 4 Discussion

The GRABS trial evaluates whether TRZ, combined with the SOC utilized 12 months after RYGB, produces greater weight loss than SOC post‐RYGB alone in adults with persistent obesity. Its premise is that obesity care lacks a defined treatment target towards which therapy is escalated, a framework long established for hypertension, dyslipidemia, and type 2 diabetes but absent in obesity. The GRABS trial addresses this gap by pairing an effective operation with an effective pharmacotherapy in patients who responded to surgery as expected yet have persistent obesity, testing whether a treat‐to‐target, combination strategy can move a meaningful proportion of such patients towards complete remission of obesity. This proactive, adjunctive model contrasts with the prevailing practice of reserving pharmacotherapy for patients who experience suboptimal surgical weight loss [36]. The trial additionally uses the altered post‐RYGB anatomy, in which gastric emptying is largely bypassed while central pathways remain intact, to distinguish the central from the peripheral, emptying‐dependent origins of TRZ's gastrointestinal adverse effects.

Prior randomized trials of post‐operative GLP1RA therapy have addressed the different population of patients with prior RYGB experiencing suboptimal response or weight recurrence. Neither term has a standardized definition: suboptimal weight loss is most often operationalized as total weight loss below 20% (or a postoperative BMI that remains ≥ 35 kg/m^2^), and weight regain as an increase of more than 10% from nadir weight [36]. The GRAVITAS trial evaluated adjunctive liraglutide primarily to improve glycemic control in patients with persistent or recurrent type 2 diabetes after metabolic surgery, rather than as a weight‐loss intervention [20]. BARI‐OPTIMISE and BARI‐STEP both enroled patients with less than 20% total body weight loss from surgery; the former tested liraglutide 3.0 mg [21], the latter, more recently, semaglutide 2.4 mg [22]. Each study enroled patients with an inadequate surgical response, positioning pharmacotherapy as a rescue. GRABS instead enrols patients with persistent obesity (BMI: ≥ 30 kg/m^2^) despite an expected surgical response, testing pharmacotherapy as a planned adjunct rather than a remedy for surgical failure. TRZ is initiated at least 12 months postoperatively, a timing informed by our retrospective data showing lower odds of GLP1RA–associated adverse events when therapy begins 12 or more months after surgery rather than earlier [23].

If TRZ augments weight loss in this setting, GRABS will provide the preliminary evidence needed to design and implement trials that define and test explicit treatment targets; for example, a target BMI below 30 kg/m^2^ versus below 35 kg/m^2^, or weight reduction continued until remission of a specific comorbidity, such as hypertension, is achieved. Demonstrating that an effective drug and an effective operation can be combined in pursuit of a predefined goal is the first step towards a target‐driven framework for obesity, in which the intensity of therapy is matched to a clinically meaningful endpoint rather than applied reactively.

The GRABS trial also begins to address a question that has not been examined in patients with prior bariatric surgery: whether GLP1RA therapy must be continued indefinitely to preserve its benefit. Obesity is a chronic, relapsing disease, and discontinuation of pharmacotherapy after an initial response is commonly followed by weight recurrence, as observed after TRZ withdrawal in the SURMOUNT‐4 trial [37, 38]. By following participants for 24 weeks after TRZ is stopped in the intervention arm, the trial explores whether weight loss achieved during combined treatment can be maintained by the durable anatomic and physiologic effects of RYGB. Evidence that weight loss is maintained off therapy would have substantial implications for the cost and feasibility of combination treatment; evidence of recurrence would reinforce that obesity, like other chronic diseases, requires sustained management.

Two features of the trial's conduct warrant comment. The trial was conducted open‐label because this trial was conducted without industry support and because no commercially available placebo matches the proprietary TRZ auto‐injector. This introduces potential bias in patient‐reported outcomes and adherence, and it created a practical recruitment problem, as four participants withdrew prior to the first visit upon learning they had been assigned to the standard‐of‐care arm. To address this, the protocol was amended to allow control‐arm participants to cross over to the experimental arm after their initial 24‐week observation period, with both arms followed for a total of 48 weeks. This amendment did not alter the primary or secondary outcome measures, which are assessed over the first 24 weeks of treatment in each arm, but it precludes a between‐arm comparison during the second 24 weeks and therefore limits the exploratory assessment of weight maintenance after TRZ discontinuation.

Enrolment was restricted to patients with prior RYGB, excluding sleeve gastrectomy (SG), for three reasons. First, the two operations produce different anatomic and physiologic states, including different postoperative incretin profiles and different regulation of gastric outflow, which involves the pylorus after SG but is bypassed after RYGB [29, 30]. Whether these differences modify the response to exogenous GLP‐1 and GIP receptor agonism is unknown, and a trial of 30 participants could neither stratify on procedure nor adjust for it. Second, the mechanism by which GLP1RAs delay gastric emptying, vagally‐mediated inhibition of antral contractility with increased pyloric tone [26, 27], remains anatomically available after SG but not after RYGB, so a mixed‐procedure cohort risked procedure‐dependent intolerance and differential attrition that would confound rather than inform the primary comparison. Third, restriction to RYGB creates the dissociation of gastric emptying‐dependent GI adverse effects and centrally mediated pro‐emetic receptor agonism, which a mixed cohort would obscure. This restriction carries direct implications for generalizability, and the present results should not be extrapolated to patients who undergo SG. While SG remains the most commonly performed metabolic and bariatric operation in the United States and worldwide, its use has been decreasing [39].

This trial has several limitations. It is a single‐centre proof‐of‐concept with a small sample (*n* = 28 after withdrawal); although adequately powered for the primary weight‐loss endpoint, its size limits generalizability and precludes definitive inference on the secondary and exploratory outcomes, which are descriptive and hypothesis‐generating. Similarly, the cohort was 82% female, and the small number of men enroled precludes assessment of heterogeneity of treatment effect by sex and limits generalizability to men. The 24‐week treatment period constrains the assessment of maximally possible weight loss and long‐term weight‐loss maintenance achieved with post‐operative TRZ. Comparison of TRZ initiated at 12 versus 18 months after RYGB is constrained by the trial's design. Although allocation to immediate or delayed initiation was randomized at entry, the delayed group begins treatment from a different postoperative weight trajectory, is observed over a non‐concurrent 24‐week period, and was subject to attrition occurring after allocation disclosure. This comparison is therefore exploratory and cannot establish the effect of timing of initiation on treatment effectiveness. Withdrawal following allocation disclosure was differential by arm (4 of 15 control participants vs. 1 of 18 assigned to TRZ); because no baseline assessments were completed, these participants cannot be fully characterized, though no further withdrawals occurred after the protocol was amended to permit crossover. Because the trial is open‐label, the co‐primary gastrointestinal symptom endpoint relies on unblinded patient‐reported outcomes and is susceptible to expectation bias, and the trial is not powered for this endpoint; the PAGI‐SYM comparison should therefore be regarded as hypothesis‐generating. Additionally, eligibility was defined by BMI, despite growing efforts to adopt other measures of central adiposity and cardiometabolic risk [40]. Finally, this study was not sufficiently long in length, nor sufficiently powered to investigate the end‐organ effects of enhanced weight loss achieved by the intervention, such as improvements in cardiac or renal function. These constraints are appropriate to a feasibility study whose purpose is to refine the intervention and estimate parameters for a definitive multicentre trial.

## 5 Conclusion

The GRABS proof‐of‐concept trial will provide the first randomized data on TRZ as a planned adjunct to RYGB in patients with persistent obesity despite an expected surgical response. By pairing an effective operation with effective pharmacotherapy to achieve a defined target, this trial offers an early test of a treat‐to‐target approach for obesity, analogous to the goal‐directed frameworks used for diabetes and hypertension. Should the combination prove effective and well‐tolerated, GRABS will yield the rationale and the design parameters for the definitive multicentre trials needed to determine whether multimodal, target‐driven care can achieve durable remission of obesity and related conditions.

## Funding

This work was supported by the National Institute of Diabetes and Digestive and Kidney Diseases of the National Institutes of Health under award number K23DK143312 (PI: Samuels). REDCap is supported by the Vanderbilt Institute for Clinical and Translational Research (VICTR) grant support (Clinical and Translational Science Award [CTSA] award number UL1 TR000445 from the National Center for Advancing Translational Sciences/National Institutes of Health). Study data were collected and managed using REDCap electronic data capture tools hosted at Vanderbilt University Medical Center. This study was additionally supported by VICTR through CTSA award number UL1 TR002243. The Vanderbilt Diabetes and Research Training Center funding (Vanderbilt Diabetes Research Center (NIH grant DK20593)) and the Vanderbilt Digestive Disease Research Center (P30 DK058404). The content is solely the responsibility of the authors and does not necessarily represent the official views of the NIH.

## Conflicts of Interest

The authors declare no conflicts of interest.

## Supporting information


**Table S1:** Baseline demographic and anthropometric measurements by arm.

## Data Availability

The data that support the findings of this study are available from the corresponding author upon reasonable request. At the conclusion of the study, per NIH requirements, upon final trial manuscript publication and no later than the end of the project period, we will archive all data, metadata, and study protocols after removing patient identifiers in the Open Science Framework (OSF) data repository.
