# Supplementary material for: GLP‐1 Receptor Agonists Post‐Bariatric Surgery (GRABS) Trial: Rationale, Design and Baseline Characteristics
Source: Diabetes Obes Metab. 2026 Sep 8;28(11):9861–70. doi: 10.1111/dom.71320 (PMC13646343; doi:10.1111/dom.71320)
Supplement: Supplementary file 1 [file DOM-28-9861-s001.docx]

Supplemental Table 1: Baseline demographic and anthropometric measurements by arm.

|  | Total (n= 33) | Intervention (n=18) | Control (n=15) |
| --- | --- | --- | --- |
| Age (years) (mean (SD)) | 42 (8.1) | 41 (7.5) | 42 (9.1) |
| Female (%) | 27 (82) | 15 (83) | 12 (80) |
| **Race** | | | |
| White (%) | 25 (75) | 13 (72) | 12 (80) |
| Black | 8 (24) | 5 (27) | 3 (20) |
| Other | 0 | 0 | 0 |
| Weight on day of Surgery (kg) (mean (SD)) | 135.4 (24.3) | 130.9 (26.6) | 137.4 (22.1) |
| BMI on day of Surgery (kg/m^2^) (mean (SD)) | 47.9 (7.6) | 48.3 (8.0) | 46.3 (7.4) |
